# Effect of provision of home-based curative health services by public sector health-care providers on neonatal survival: a community-based cluster-randomised trial in rural Pakistan

**DOI:** 10.1016/S2214-109X(17)30248-6

**Published:** 2017-07-14

**Authors:** Sajid Soofi, Simon Cousens, Ali Turab, Yaqub Wasan, Shah Mohammed, Shabina Ariff, Zaid Bhatti, Imran Ahmed, Steve Wall, Zulfiqar A Bhutta

**Affiliations:** a Centre of Excellence in Women and Child Health, Aga Khan University, Karachi, Pakistan; b London School of Hygiene & Tropical Medicine, London, UK; c Saving Newborn Lives Program, Save the Children, Washington, DC, USA; d Centre for Global Child Health, The Hospital for Sick Children, Toronto, ON, Canada

## Abstract

**Background:**

Although the effectiveness of community mobilisation and promotive care delivered by community health workers in reducing perinatal and neonatal mortality is well established, evidence in support of home-based neonatal resuscitation and infection management is mixed. We assessed the effectiveness of adding training in neonatal bag and mask resuscitation and oral antibiotic therapy for suspected neonatal infections to a basic preventive and promotive interventions package delivered by public sector community-based lady health workers (LHWs) in rural Pakistan.

**Methods:**

We did a cluster-randomised controlled trial in two subdistricts of Naushahro Feroze in rural Sindh, Pakistan, between April 15, 2009, and Dec 10, 2012. LHWs, trained in basic newborn resuscitation and in recognition and treatment (with oral amoxicillin) of suspected neonatal respiratory infections, were linked with traditional birth attendants and encouraged to attend home births. Control clusters received routine care through the existing national programme. The primary outcome was all-cause neonatal mortality. Independent data collection teams recorded data for all pregnancies and their outcomes, morbidity, mortality, and household practices related to maternal and newborn care.

**Findings:**

Of the 27 randomised clusters with functional LHW programmes, 13 were allocated to the intervention group (n=242 749) and 14 to the control group (n=256 985). In the intervention group, LHWs did 80% of the planned community mobilisation sessions, but were able to attend only 1184 (14%) of 8425 deliveries and 4318 (25%) of 17 288 neonatal visits within 72 h of birth (p<0·0001 for both variables compared with the control group). The neonatal mortality rate was 42 deaths per 1000 livebirths in intervention clusters compared with 55 per 1000 in the control group (risk ratio 0·80, 95% CI 0·68–0·93; p=0·005).

**Interpretation:**

The reduction in neonatal mortality in intervention clusters occurred against a background of improvements in domiciliary practices for maternal and newborn care. However, the poor reach of LHWs in accessing newborn infants at birth and in the early postnatal period underscores the limitations of tasking community health workers in public sector programmes working in similar circumstances with such complex interventions. Such community-based interventions in health systems should be accompanied by concerted efforts to improve quality of care in facilities and referral systems.

**Funding:**

Saving Newborn Lives, Save the Children USA.

## Introduction

Globally, 5·9 million children still die yearly before reaching their fifth birthday.^1^ 2·7 million of these deaths are accounted for by neonates.^2^, ^3^ Much of the improvement in child survival over the past few decades has resulted from reductions in post-neonatal deaths from measles, pneumonia, and diarrhoea.^4^ Other than a reduction in deaths due to neonatal tetanus, improvements in neonatal survival in much of south Asia have lagged behind reductions in post-neonatal mortality.

About two-thirds of the global burden of maternal deaths, neonatal deaths, and stillbirths is concentrated in ten large countries in sub-Saharan Africa and south Asia, of which Pakistan is one.^5^, ^6^ Two groups of conditions account for most neonatal deaths: preterm birth complications (an estimated 1·055 million deaths) and intrapartum-related events, which were formerly known as birth asphyxia (0·691 million deaths).^1^ A further 0·581 million neonatal deaths annually are due to sepsis, meningitis, pneumonia, and diarrhoea.^1^ Almost half of all stillbirths occur during labour and delivery, and nearly half of all neonatal deaths occur in the hours immediately after birth.^5^ Although the proportion of facility-based births is increasing in many countries, a large proportion of deaths occur at home, and combinations of community-based services, outreach services, and high-quality facility-based services are needed to make a difference.^7^, ^8^ In view of shortages of trained physicians and midwives in many settings, task shifting to a range of ancillary health workers, including community health workers, is a possible option.^9^

**Research in context**

**Evidence before this study**

The role of community-based approaches and community health workers in promotion of care and delivery of health-care messages to improve perinatal and newborn care is well established. We did a systematic review of available information about community-based strategies for improving newborn care with community health workers in Pakistan, and also consulted the global literature. We searched PubMed, the Cochrane library, and regional databases of WHO and UNICEF with the terms “community health workers” or “community platforms” and “newborn”, and linked these medical subject heading terms to “Pakistan” to identify articles published in English on or before March 31, 2017. A Cochrane review included data from 26 cluster-randomised or quasi-randomised trials of a wide range of interventional packages, including two subsets from three trials. The data showed major reductions in neonatal mortality (including both early and late mortality), stillbirths, and perinatal mortality as a result of implementation of community-based interventional care packages. Although we identified strong evidence of improved household behaviours and improved care seeking in facilities, results for home-based neonatal resuscitation by either community health workers or traditional birth attendants were mixed, and results for antibiotic administration by community health workers were limited to a few efficacy trials, none of which were done in large public sector programmes. After completing a feasibility assessment, we worked with the National Program for Family Planning and Primary Care in Pakistan to assess the effect of training lady health workers (LHWs) in rural Pakistan to attend births, provide home-based bag and mask resuscitation as required, and provide oral amoxicillin to neonates with suspected pneumonia or serious infections before referral.

**Added value of this study**

Our cluster-randomised trial showed that community intervention by the LHW programme led to a 20% reduction in neonatal mortality and was associated with significant improvements in household practices and newborn care practices. However, overall effective coverage by functional LHWs was only 48%, and they were able to perform resuscitation in only 4% of potentially eligible neonates with birth asphyxia. The intervention had no effect on cause-specific neonatal mortality due to asphyxia or suspected serious infections. We also identified no effects on care seeking for facility births and stillbirths in the intervention clusters.

**Implications of all the available evidence**

Our trial supports the use of community health workers in large programmes for community mobilisation and support strategies for preventive and promotive maternal and newborn care. However, in view of the reality of large-scale public sector programmes, tasking such health workers with complex additional domiciliary care responsibilities might not be advisable. Improvement of maternal and newborn care in facilities and promotion of care seeking and transportation could prove more effective.

In Pakistan, despite some improvements in coverage of antenatal care and skilled attendance, a high proportion of neonatal deaths occur at home, particularly in rural areas, where few trained professionals and skilled birth attendants are available.^10^, ^11^ Results of a 2012–13 survey of demographics and health showed large urban–rural disparities in terms of delivery (32% of deliveries in urban areas were home births *vs* 60% in rural areas) and neonatal mortality (47 deaths per 1000 livebirths in urban areas *vs* 62 per 1000 in rural areas).^12^ The Pakistani Government initiated a rural health programme with community health workers—so-called lady health workers (LHWs)—in 1994, with a focus on preventive and promotive strategies for maternal health, family planning, and primary care.^11^ More than 100 000 LHWs are deployed across rural Pakistan, but coverage is variable (ranging from 40–80%),^13^ and they do not routinely attend home deliveries. Each LHW is responsible for maintenance of birth records, provision of a range of promotive and preventive educational services, management of milder illnesses such as childhood diarrhoea and respiratory infections, and referral of people who need high-level care to health facilities for about 100 households in rural villages. They also provide services for family planning, basic maternal antenatal care, and oral polio vaccines during vaccination campaigns, and promote routine immunisations.^14^

Several community-based trials have been done in rural Pakistan to assess the potential effects of training community health workers on neonatal mortality. These trials included both public sector LHWs and community health workers supported by non-governmental organisations to deliver community mobilisation, health education through home visits,^15^, ^16^ and innovations such as the use of cord chlorhexidine by traditional birth attendants.^17^ We have also shown that strengthening of the LHW programme's links with the community and promotive care through community group sessions is associated with reductions in perinatal and neonatal mortality.^15^ Although other community-based strategies—eg, women's groups,^18^ promotion of preventive interventions such as exclusive breastfeeding—improved neonatal outcomes in low-income and middle-income countries,^19^, ^20^ the success of therapeutic interventions such as neonatal resuscitation and antibiotics has varied.^21^, ^22^, ^23^, ^24^ Community-based management of pneumonia and severe pneumonia in children older than 1 month by LHWs was effective in rural Pakistan,^25^, ^26^ but effectiveness in possibly infected neonates has not been fully assessed. Thus, despite long-standing recommendations to increase the range of interventions to improve neonatal survival in settings where referral is difficult or not possible,^27^ the effectiveness of home-based management of neonates in need of resuscitation at birth or born prematurely by front-line community health workers in programmatic settings is unclear. If effective, such strategies could have clear benefits, including reducing neonatal mortality, in populations with little access to doctors, nurses, or midwives.

In collaboration with Pakistan's national programme for family planning and primary care, we did a cluster-randomised effectiveness trial of training LHWs to deliver a preventive and promotive community mobilisation and education package^15^ alongside recognition of possibly asphyxiated newborn infants at birth and bag and mask resuscitation as needed, and recognition and management of suspected neonatal infections.

## Methods

### Study setting and development

We did a prospective cluster-randomised trial between April 15, 2009, and Dec 10, 2012, in the district Naushahro Feroze in rural Sindh. The district is located 450 km north of Karachi and has five *talukas* (subdistricts) and an official population of around 1·3 million. The trial was done in a subpopulation of 0·56 million in two *talukas* of Naushahro Feroze (Bhirya [population 0·26 million], and Naushahro Feroze [population 0·23 million]) and three *talukas* of Moro and Kandiaro (combined population 0·07 million). The study site is typical of most rural districts in Sindh and southern Punjab. The trial was approved by the ethics review committee of the Aga Khan University (1212-Ped/ERC). All households in the selected districts were included in the study. Community assent was obtained from village representatives for participation in the study; participating women gave verbal consent.

To develop the intervention and define the clusters, we did a baseline cross-sectional household survey of the catchment population of 35 health facilities (14 basic health units, 12 government dispensaries, eight rural health centres, and the referral district headquarters hospital) between April 15 and Aug 30, 2009. We collected information about knowledge and practices relating to neonatal care from a random sample of newly delivered mothers identified in the baseline survey. We then did a formative qualitative study to develop and adapt the proposed intervention package to the local context and assess acceptability in close consultation with the federal and provincial LHW programmes and health departments. The preventive component of the package was adapted from one used in a previous trial.^14^ The therapeutic components of the intervention package focused on the immediate household management of intrapartum events (birth asphyxia), recognition of low birthweight and suspected serious neonatal infections, and prompt referral to public sector hospitals.

We did a planned pilot trial of improved practices between July 1 and Dec 31, 2009, in the catchment population of one health facility, which was subsequently excluded from the main trial. The study tested the package, refined implementation, and assessed the feasibility of linking LHWs with traditional birth attendants so that LHWs could attend home births, and the ability of LHWs to use a bag and mask for neonatal resuscitation. On the basis of this pilot trial, the intervention package was finalised before rollout in the main trial (table 1).

**Table 1:** Description of intervention package

	**Intervention clusters**	**Control clusters**
**LHWs' programme of support and training**		
Recognition of high-risk pregnancies and neonatal danger	Yes	Yes
Promotion of antenatal care and use of iron or folate in pregnancy	Yes	Yes
Promotion of adequate maternal diet and rest	Yes	Yes
Provision of clean delivery kit to pregnant women	Yes	No
Immediate neonatal care	Yes	Yes
Promotion of exclusive and early breastfeeding	Yes	Yes
Cord care (dry, clean, and avoid any traditional application)	Yes	Yes
Delayed bathing	Yes	Yes
Recognition and domiciliary care of neonates with birth asphyxia, low birthweight, and suspected sepsis, and referral	Yes	No
LHWs present at home births	Yes	No
Domiciliary care with bag and mask for asphyxiated neonates and referral for aftercare	Yes	No
Improved thermal care for low-birthweight and premature babies (frequent breastfeeding, waddling, co-bedding, early referral in case of any danger sign)	Yes	No
Provision of first dose of amoxicillin to suspected infected neonates and referral to referral hospital; daily follow-up and provision of amoxicillin for 7 days in case of refused referral to hospital	Yes	No
Provision of inflatable bag and mask, sucker bulb, amoxicillin, clean delivery kits, and management protocols to LHWs	Yes	No
**Support group (health education) training**		
Exclusive training on support group methods, communication, and counselling skills for LHWs	Yes	No
Male motivators training	Yes	No
Incorporation of three flip charts on birth asphyxia, low birthweight, and sepsis in LHW curriculums	Yes	No
**Orientation for traditional birth attendants (*dais*)**		
Basic essential neonatal care training and linkage with LHWs	Yes	No
**Health facility strengthening**		
Health-care providers training on essential neonatal care and management of birth asphyxia, low-birthweight babies, and neonatal sepsis	Yes	No
Health-care providers training on essential neonatal care and management of sick newborn infants according to WHO guidelines	No	Yes
Provision of inflatable bag and mask and oral amoxicillin with management protocols	Yes	No

LHW=lady health workers.

### Cluster definition, randomisation, and masking

We defined a cluster as the catchment population of an individual functional primary care facility (basic health units and rural health centres) and their affiliated LHWs. Basic health units typically serve a population of 10 000–20 000 and have 10–20 affiliated LHWs. Rural health centres cater to a population of 25 000–50 000 and have 25–50 affiliated LHWs. Most LHWs have a catchment population of about 1000 individuals (120–200 households), are mostly resident in the same area, and are not transferred to other facilities or areas. In the original trial proposal, we anticipated the potential inclusion of 34 clusters representing the entire district.

Clusters were assigned (1:1) to either the intervention or control groups. To ensure reasonable balance between the two arms, we used stratified, restricted randomisation to allocate clusters. Two strata were defined on the basis of level of health facilities: hospital or rural health centre (nine clusters) or basic health units (18 clusters). 1 million random allocation schemes were generated by the study statistician (SC), who used a computer algorithm. Acceptable schemes were those in which the total populations of each arm was restricted to within 15 000 of each other, total livebirths per year per arm to within 1000 of each other, overall neonatal mortality rates to within five per 1000 livebirths of each other, the ratio of LHWs to population to be within one per 10 000 population of each other, and overall female literacy rates to be within 5% of each other. In all, 28 476 distinct allocation schemes satisfying these restriction criteria were available, of which one was randomly chosen by the algorithm, with random allocation of one of the arms to the intervention.^28^ Delivery of the intervention was not blinded for practical reasons. Data collection teams were not actively informed which clusters were allocated to intervention and control arms.

### Procedures

The trial was fully integrated and implemented within a programmatic setting. Senior faculty members of the Division of Women and Child Health of Aga Khan University (Karachi, Pakistan) held 5 days' training for LHW programme master trainers, who subsequently trained LHWs from the intervention clusters at the health facilities to which they were affiliated (an initial 3 days of training and monthly 1 day refresher sessions thereafter). Study supervisors along with LHW programme managers monitored the refresher sessions. Each intervention LHW was provided with a bag and mask for neonatal resuscitation (Laerdal Medical, Stavanger, Norway) and oral amoxicillin (125 mg/1·25 ml, to be given as 50 mg/kg per dose). LHWs were also given pictorial guides describing the management of asphyxia, thermal care, co-bedding, breastfeeding of low-birthweight babies, and recognition of suspected pneumonia and administration of oral amoxicillin before referral.

As already included in the LHW programme guidance, we reinforced the importance of linkages of LHWs with traditional birth attendants in their areas. LHWs were encouraged to maintain close links with traditional birth attendants, keep records of expected births, and attend home deliveries. Clean delivery kits were provided to pregnant women in the intervention clusters during health education sessions delivered by LHWs, and the importance of provision of urgent neonatal care at birth, if needed, was emphasised. LHWs were trained as per national and project guidelines to do additional postnatal visits on days 3, 7, 14, and 28 after birth. LHWs were reimbursed additional travel costs, if any, to attend deliveries or postnatal visits, but no additional salary or other financial incentives were provided. A 3-day orientation programme in basic immediate maternal and newborn care was also organised for traditional birth attendants in the intervention arm, who were trained in the use of clean delivery kits, and strongly encouraged to inform LHWs in a timely manner to attend the home birth. No remuneration, commodities, or resuscitation training was provided to traditional birth attendants.

Separate training sessions on health education and community mobilisation were held for male community mobilisers—volunteers from the villages, who were tasked with monthly mobilisation and review meetings with male elders and members of the community. These meetings aimed to promote antenatal care, postnatal care, and facility births. For people choosing to deliver at home, the importance of informing the LHW so that she could attend the childbirth was also reinforced, and volunteers were identified to escorts LHWs to attend deliveries, especially after dark.

In the control clusters, the LHW programme continued to function as usual. LHWs continued to have regular monthly debriefing and refresher trainings according to the standard national LHW programme curriculum in the health facilities to which they were affiliated. However, as in the intervention arm, health-care providers from public sector facilities in the entire district received a one-time refresher training on essential neonatal care and management of sick newborn infants according to WHO guidelines^29^ in three separate workshops done between January and May, 2010.

LHW programme supervisors monitored the delivery of the intervention package and related components and maintained their own records. An independent surveillance system was implemented, with 13 data collection teams visiting each household in the trial area quarterly. Verbal consent from heads of households and respondents was obtained for data collection. These data collectors were managed and deployed independently of the LHW programme and shuffled periodically as per previous surveillance protocols.^14^ They gathered standardised information from each household on all pregnancies, their outcomes, new pregnancies, neonatal morbidity and mortality, in-migrations, and out-migrations. Data collectors recorded whether the LHW was present at the time of delivery and instituted any interventions on the baby, and recorded treatments provided and referrals to hospital. Every 6 months, women reporting a livebirth since the previous surveillance visit were interviewed with a structured questionnaire to assess knowledge and practices related to neonatal care and LHW visits or actions.

In addition to their official logbooks, LHWs in the intervention clusters were encouraged to record and maintain information about home visits, neonatal illnesses, management of babies with breathing difficulties, low birthweight, and suspected serious infections, referrals, and outcomes on separate forms provided by the research team. The research team monitored the monthly LHWs refresher training meetings and reviewed the forms provided by LHWs. Verbal autopsies of all stillbirths and neonatal deaths were done by a separate team of trained anthropologists within 2–16 weeks of the event with standard WHO-recommended instruments.^30^, ^31^

### Outcomes

The primary outcome of the trial was all-cause neonatal mortality. The initial proposal included perinatal mortality as a primary outcome, but on the basis of feedback from the LHW programme and the range of outcomes captured therein, we principally focused on neonatal mortality instead. Secondary outcomes included cause-specific neonatal mortality due to intrapartum events, prematurity, and sepsis, and the stillbirth rate.

### Statistical analysis

In the Hala trial,^13^ an almost 20% reduction in neonatal mortality was reported with LHW training in preventive care and community mobilisation for improved household practices and care seeking compared with the control population, and thereafter the LHW programme adopted the Hala package within its training programme. In view of the 45–50% reduction in mortality reported in other trials of home-based treatment,^21^, ^32^, ^33^ we estimated that a 40% reduction in neonatal mortality from the enhanced intervention was plausible. We assumed an average cluster size of 10 000, a crude annual birth rate of 25 per 1000, and an average neonatal mortality rate of 40 per thousand livebirths in the control arm (coefficient of variation 0·25). We estimated that inclusion of all births for 3 years would provide greater than 90% power to detect a 40% reduction in neonatal mortality rate and close to 80% power to detect a 30% reduction.^28^

We compared baseline characteristics of intervention and control areas by visual inspection. During the intervention period, frequency of birthing practices, LHW contacts, and neonatal morbidity rates in both groups were compared with logistic regression, with robust SEs to account for between-cluster variation, and adjusted for surveillance round and randomisation stratum. Analysis of mortality outcomes was done with generalised estimating equations, with robust SEs to account for the cluster randomisation. To obtain estimates of the risk ratio for intervention versus control clusters, binomial regression models with a log link were fitted, controlling for cluster-level log(baseline mortality, stillbirths, and early pregnancy loss), randomisation stratum, and surveillance round. The trial is registered with ClinicalTrials.gov (NCT01350765).

### Role of the funding source

Although SW was involved in periodic review of the study progress, the funder of the study had no role in study design; data collection, analysis, or interpretation; or writing of the report. The corresponding author had full access to all the data in the study and had final responsibility for the decision to submit for publication.

## Results

**Figure 1:**
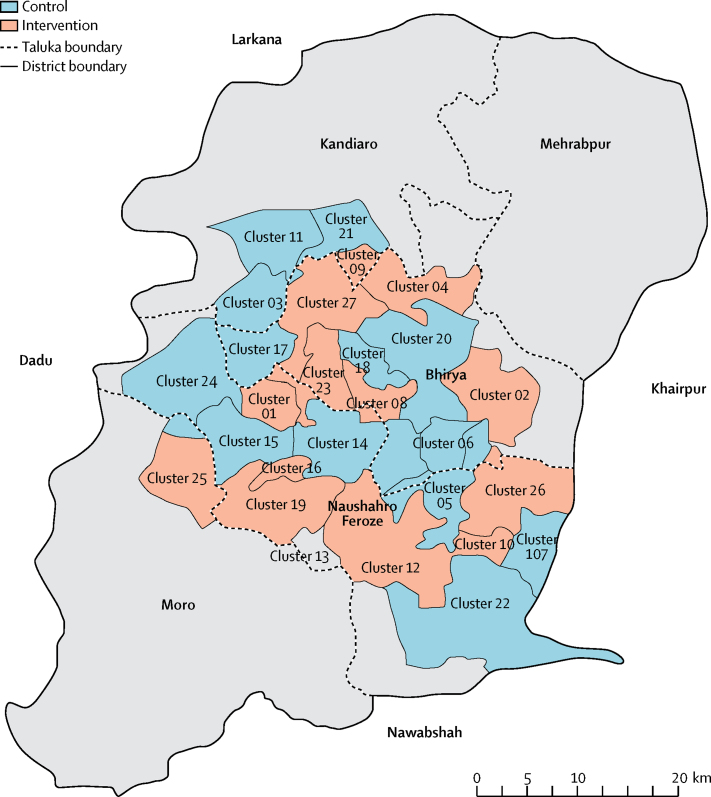
Study site and clusters

**Figure 2:**
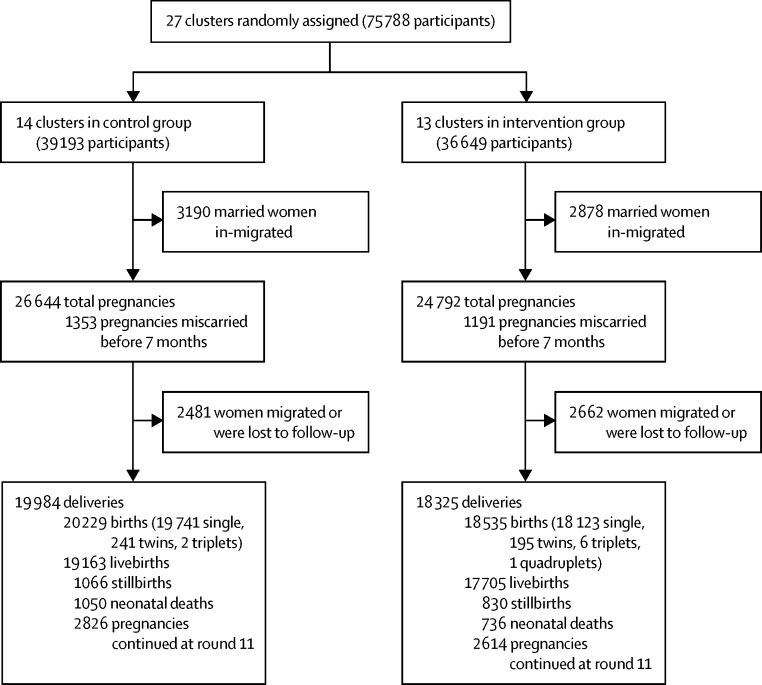
Trial profile

The trial was planned to span 36 months, but ended in Dec 10, 2012 (after quarterly surveillance round 11), because of the conclusion of activities in Pakistan by the Saving Newborn Lives programme of Save the Children, USA. Seven of the 34 clusters in the study area could not be included because no LHWs were posted therein. Thus 27 clusters, including 35 health-care facilities and their affiliated LHWs, were included (Figure 1, Figure 2). The entire population of the 27 clusters was included in the trial.

At baseline, the study groups were balanced in terms of population and number of households (table 2). 73% of women in both groups had no education (table 2). Most households owned their homes (96%), had electricity (96%), and had access to piped water (88%; table 2). Roughly a quarter of the households in both groups had no toilets; only 19% of houses in control villages had underground waste disposal arrangements, compared with 10% in the intervention areas (table 1). According to the baseline survey, neonatal mortality and stillbirth were slightly less frequent in the intervention clusters than in the control clusters (43·7 *vs* 44·5 per 1000 livebirths, and 39·4 *vs* 42·7 per 1000 total births, respectively; table 2). The rate of early fetal death at baseline was broadly similar in both groups (table 2), as were the major causes of stillbirths and neonatal deaths (appendix).

**Table 2:** Baseline sociodemographic characteristics

		**Control (14 clusters)**	**Intervention (13 clusters)**
Population		256 985	242 749
Households		32 498	30 959
People per household		7·91 (4·30)	7·84 (4·81)
Children younger than 5 years per household		1·41 (1·23)	1·32 (1·19)
Female education			
	n	51 806	49 192
	No education	37 578 (73%)	36 045 (73%)
	Religious education only	4855 (9%)	3846 (8%)
	Primary and middle school	6254 (12%)	6005 (12%)
	Higher secondary school	2434 (5%)	2616 (5%)
	Secondary school graduate and above	685 (1%)	680 (1%)
Finished walls (cement, stone with lime, or bricks)		8119 (25%)	7412 (24%)
Finished floor (ceramic tiles, cement, or carpet)		6697 (21%)	5806 (19%)
Finished roofing (ceramic tiles, cement, or roofing shingles)		10 455 (32%)	9484 (31%)
Families owning their own home		31 177 (96%)	29 650 (96%)
Single-room households		18 319 (56%)	16 756 (54%)
Households with piped water		28 163 (87%)	27 433 (89%)
Households with no toilet facility		8072 (25%)	7044 (23%)
Households with underground sewerage (liquid waste)		6095 (19%)	3188 (10%)
Households with electricity		31 182 (96%)	29 871 (96%)
Households using solid fuels for cooking		25 796 (79%)	23 953 (77%)
Birth outcomes and mortality rates			
	Livebirths	8071	7755
	Stillbirths	360	318
	Early fetal deaths	1016	826
	Neonatal deaths	358	339
	Neonatal deaths per 1000 livebirths	44·6	43·7
	Stillbirths per 1000 births	42·7	39·4
	Early fetal death rate per 1000 pregnancies	107·6	92·8

Data are n, n (%), or mean (SE).

Of the 63 457 households in the study area, 35 155 (55%) were included in the LHW programme registers and hence covered. Of the 272 officially appointed LHWs in the intervention clusters, only 228 (84%) were fully functional and working (18 did not engage with the project, 12 were non-residents of the area, eight had not received formal training, and six were transferred to the study areas during the study; table 3). During the intervention period, LHWs reported doing 6943 (80%) of the 8650 planned female health education sessions in intervention villages. At different periods, together representing almost a third of the overall length of the trial, LHWs were involved in local polio campaigns and mother and child activities, and were hence unavailable for trial-specific activities. 192 LHWs (71%) did more than 20 community sessions during the intervention period (table 3).

**Table 3:** LHW characteristics and programme performance

	**n (%)**
**LHWs in intervention clusters**	
Transferred from other health-care facility	6 (2%)
Non-resident	12 (4%)
Uncooperative	18 (7%)
Untrained	8 (3%)
Functional	228 (84%)
**Health education sessions done by LHWs**	
0	8 (3%)
1–10	29 (11%)
11–20	43 (16%)
>20	192 (71%)
**Neonates visited by individual LHWs**	
0	11 (4%)
1–33	147 (54%)
34–66	100 (37%)
>66	14 (5%)

N=272. Overall, 6943 health education sessions were done, and 8471 neonates were visited. LHW=lady health workers.

Between February, 2010, and December, 2012, the actual period of trial intervention, 51 436 pregnancies were identified in the trial area (appendix). 2544 pregnancies (5%) ended in early fetal death (<28 weeks' gestation), 5143 women (10%) migrated out of the study area, and 5440 women (11%) were still pregnant at the end of the trial (appendix). We noted no significant differences between the intervention and control clusters in the proportions of facility births (53% *vs* 54%; p=0·73) and deliveries facilitated by skilled birth attendants (54% *vs* 56%; p=0·53; table 4). A significantly higher proportion of home deliveries were attended by LHWs in the intervention clusters than in the control clusters (14% *vs* 1%; p<0·0001; table 4). Clean delivery kits were used significantly more often for home deliveries in the intervention clusters than in the control clusters (p<0·0001; table 4).

**Table 4:** Post-intervention birthing practices and neonatal morbidity

	**Control clusters**	**Intervention clusters**	**OR (95% CI)**	**p value**
Home births	9238/19 984 (46%)	8627/18 325 (47%)	1·04 (0·82–1·34)	0·73
Facility births	10 746/19 984 (54%)	9698/18 325 (53%)	0·96 (0·75–1·22)	0·73
Skilled birth attendant present	11 150/19 984 (56%)	9900/18 325 (54%)	0·92 (0·72–1·18)	0·53
Presence of LHW at deliveries managed by traditional birth attendants	55/8834 (1%)	1184/8425 (14%)	28·4 (14·3–56·4)	<0·0001
Instrumental deliveries	3474/19 984 (17%)	3008/18 325 (16%)	0·94 (0·81–1·09)	0·42
Use of clean delivery kits at home births	1488/7723 (19%)	4236/7698 (55%)	6·16 (2·91–13·0)	<0·0001
LHW postnatal visits	271/18 609 (1%)	5256/17 288 (30%)	32·3 (15·7–66·1)	<0·0001
LHW early postnatal visits	188/18 609 (1%)	4318/17 288 (25%)	35·5 (17·9–70·7)	<0·0001
Neonates with reported breathing problem or delayed cry at birth	2825/19 163 (15%)	2391/17 705 (14%)	0·90 (0·72–1·12)	0·34
Neonates with breathing problem resuscitated by LHW	2/2825 (<1%)	98/2391 (4%)	67·6 (14·2–320·5)	<0·0001
Low-birthweight births	992/10 159 (10%)	933/10 125 (9%)	0·96 (0·76–1·22)	0·74
Neonates with reported illness	6626/19 163 (35%)	5439/17 705 (31%)	0·84 (0·61–1·14)	0·26
Care seeking for neonates with reported illness	6137/6626 (93%)	5037/5439 (93%)	1·02 (0·76–1·37)	0·89
Sick neonates visited by LHW	102/6626 (2%)	1566/5439 (29%)	27·2 (13·5–54·5)	<0·0001
Neonates with possible infection	5201/19 163 (27%)	4350/17 705 (25%)	0·87 (0·66–1·16)	0·36
Neonates with possible infection seen by LHW	86/5201 (2%)	1252/4350 (29%)	25·1 (13·2–47·7)	<0·0001
Neonates with possible infection managed with amoxicillin given by LHW	19/5201 (<1%)	707/4350 (16%)	54·6 (21·8–137·3)	<0·0001

Data are n/N (%). OR=odds ratio. LHW=lady health worker.

During the study period, rates of early fetal loss, although much lower than reported baseline values, were broadly similar in both groups (61 per 1000 pregnancies in intervention clusters *vs* 63 per 1000 pregnancies in control clusters; risk ratio [RR] 0·97, 95% CI 0·88–1·08; p=0·60; table 5).

**Table 5:** Summary of birth outcomes from the quarterly surveillance (rounds 1–11) by trial group

	**Control clusters**	**Intervention clusters**	Mortality risk ratio*(95% CI)	**p value**
**Early pregnancy loss**				
n	1353	1191	..	..
Rate per 1000 known pregnancies	63	61	0·97 (0·88–1·08)	0·60
**Stillbirths**				
n	1066	830	..	..
Rate per 1000 total births	53	45	0·89 (0·76–1·04)	0·13
**Early neonatal mortality**				
n	871	610	..	..
Rate per 1000 livebirths	45	34	0·79 (0·67–0·93)	0·006
**Late neonatal mortality**				
n	179	126	..	..
Rate per 1000 livebirths	9	7	0·72 (0·62–0·85)	0·0001
**Neonatal mortality**				
n	1050	736	..	..
Rate per 1000 livebirths	55	42	0·80 (0·68–0·93)	0·005
**Perinatal mortality**				
n	1937	1440	..	..
Rate per 1000 total births	96	78	0·86 (0·75–0·99)	0·03

* Estimated with generalised estimating equations, controlling for baseline mortality, randomisation stratum, and surveillance round.

Stillbirth rates were somewhat higher than those reported in the baseline survey, but again did not differ significantly between groups after baseline rates and randomisation were controlled for (RR 0·89, 95% CI 0·76–1·04; p=0·13; table 5; appendix). The neonatal mortality rate was lower in intervention clusters than in control clusters (42 *vs* 55 per 1000 livebirths; RR=0·80, 95% CI 0·68–0·93; p=0·005; table 5). In the first surveillance round after implementation of the intervention, neonatal mortality in both groups was similar, and thereafter consistently lower in the intervention clusters (appendix). An analysis of cluster-level summaries produced a broadly similar pattern of results, but with point estimates suggesting slightly larger intervention effects on mortality endpoints, particularly for stillbirths and perinatal mortality (appendix). Our study was not powered for cause-specific neonatal mortality outcomes and we identified no significant difference between the groups for major categories of neonatal mortality, including preterm birth complications, perinatal asphyxia, and neonatal infections, or for most causes of stillbirths, except for those related to obstructed labour and complications (p=0·003; appendix).

Of all the livebirths in the intervention clusters, 2391 neonates reportedly had breathing problems or delayed cry at birth, 98 (4%) of whom were resuscitated by LHWs attending births (90 survived; appendix). 4350 neonates in intervention clusters had some features of suspected infections, including fast breathing, according to maternal reports (appendix). LHWs visited 1252 (29%) of these neonates, of whom 707 (56%) were managed with oral amoxicillin (appendix). 661 (93%) given amoxicillin survived (appendix). In control clusters, LHWs visited 82 (2%) of the 5201 neonates who had signs of suspected infections. 19 (23%) of the visited were managed with oral amoxicillin, 17 (89%) of whom survived (appendix).

More women from intervention clusters (35%) than from control clusters (2%) reported visits by LHWs during the antenatal period (p<0·0001; table 6). Similar proportions of women from both groups sought antenatal care at least once during pregnancy and received two or more doses of the tetanus toxoid vaccine during the antenatal period (table 6). More women in the intervention arm than in the control arm were visited by the LHW within 3 days of delivery (29·9% versus 0·4%; p<0·0001). Breastfeeding within 1 h of birth, giving colostrum to neonates, and co-bedding or swaddling were significantly more common in intervention than in control clusters (table 6). Restriction of the analysis to functional LHWs and the covered areas only suggested that birth attendance, use of clean delivery kits, postnatal visits, and sick neonates seen were slightly higher than those in the intervention group overall, but rates of neonatal resuscitation were broadly similar (appendix). Broadly similar findings were noted in the restricted analysis comparing functional LHWs in the intervention clusters with overall findings in the intervention clusters (appendix). In this restricted analysis, 23% of neonates with possible infections were given amoxicillin (appendix).

**Table 6:** Household knowledge, attitudes, and practices around delivery at surveillance rounds 3, 5, 7, 9, and 11

	**Control clusters (n=10 859)**	**Intervention clusters (n=10 118)**	**OR (95% CI)**	**p value**
Women attending at least one antenatal consultation	8568 (79%)	8413 (83%)	1·33 (0·96–1·82)	0·08
Women attending four or more antenatal consultations	2161 (20%)	2567 (25%)	1·41 (1·07–1·87)	0·01
Women visited by LHW during pregnancy	230 (2%)	3566 (35%)	29·0 (13·3–62·7)	<0·0001
Women receiving two or more tetanus toxoid vaccination doses during pregnancy	1257 (12%)	1364 (14%)	1·15 (0·87–1·53)	0·32
Use of new blade for cutting cord	4608 (42%)	3984 (39%)	0·86 (0·69–1·09)	0·22
Use of cord clamp for tying cord	5703 (53%)	5186 (51%)	0·95 (0·76–1·19)	0·68
Cord cutting after placenta delivery	9653 (89%)	8980 (89%)	0·97 (0·63–1·50)	0·89
Dry cord care	1304 (12%)	1580 (16%)	1·35 (1·03–1·76)	0·03
Use of new cloth or clean towel for cleaning and drying neonate	6357 (59%)	6117 (61%)	1·07 (0·81–1·42)	0·61
Delayed bathing until after 6 h	6585 (61%)	6318 (62%)	1·08 (0·76–1·52)	0·68
Neonates warmed after birth	10 727 (99%)	10 021 (99%)	1·25 (0·85–1·83)	0·27
Mothers giving colostrum	6678 (61%)	7355 (73%)	1·77 (1·50–2·10)	<0·0001
Mothers starting breastfeeding within 1 h	3079 (28%)	3956 (39%)	1·65 (1·26–2·16)	0·0002
Neonates receiving skin-to-skin contact with mother (co-bedding or swaddling)	4130 (38%)	4929 (49%)	1·55 (1·15–2·12)	0·005
Mothers' awareness to take appropriate* action for asphyxiated babies	1293 (12%)	1699 (17%)	1·50 (1·22–1·81)	<0·0001
Mothers' awareness to seek care for low-birthweight babies	10 723 (99%)	9941 (98%)	0·74 (0·45–1·23)	0·25
Neonatal massage	10 105 (93%)	9633 (95%)	1·51 (1·10–2·08)	0·01
Mothers visited by LHW within 3 days of delivery	43 (<1%)	3022 (30%)	115·2 (57·7–230·1)	<0·0001

Data are n (%). OR=odds ratio. LHW=Lady health worker.

* Clean mouth and nose, rub back of baby, and give artificial respiration.

## Discussion

In our cluster-randomised study of the effectiveness of an integrated community-based package comprising preventive and home-based immediate curative care delivered by public sector LHWs in in a programmatic setting in rural Pakistan, we noted a 20% reduction in neonatal mortality in intervention compared with control clusters. This 20% reduction is similar to the 15% reduction reported in an earlier trial, which did not include additional training of LHWs to resuscitate neonates at home as required or treat suspected pneumonia with antibiotics.^15^

This overall effect of the combined intervention was much smaller than we hypothesised at trial outset, and was lower than the reductions recorded in other cluster-randomised trials^32^, ^33^ in the region. However, those studies were mostly efficacy trials that directly employed, supervised, and remunerated community health workers, as opposed to true effectiveness studies done within existing public sector health systems with their inherent constraints. In the only effectiveness trial^34^ of scale-up of integrated management of neonatal and childhood illnesses, which was done through the public health system in India, a much smaller (9%) reduction in neonatal mortality was reported. The Newhints trial^35^ in Ghana, which was based on home visits by existing community-based volunteers, showed increases in the coverage of several essential newborn care behaviours, but did not significantly affect neonatal mortality.

Our findings suggest limited additional benefits compared with basic promotive and preventive care of training public sector LHWs in Pakistan to resuscitate newborn infants delivered at home and treat suspected neonatal infections with oral antibiotics. They are also a stark reminder of the limitations to what busy public sector community health workers can deliver. In addition to community health workers' technical limitations, the effectiveness of such additional activities is dependent on the workload and range of other duties that such workers have in health systems. However, we think that the reduction in neonatal mortality shows the importance of community outreach services and the role of task shifting in reducing perinatal and neonatal mortality in such high-risk rural populations.

Several key household behaviours related to maternal and newborn care improved in the households of the intervention clusters compared with control clusters, suggesting that community-based health promotion was effective. These findings are consistent with those noted in the previous trial of LHWs in Hala,^15^ and included an important increase in routine LHW visits to mothers in both the antenatal and postnatal periods and evidence of improved practices such as colostrum administration, early initiation of breastfeeding, and dry cord care.

Our study design and operational plan had several strengths. First, the enhanced training programme was integrated with the district LHW programme and implemented like other regular trainings in the district health system. Training in newborn care was guided by specific learning objectives and accompanied by assessment of trainee performance and skills, which all LHWs passed. We achieved close engagement of the LHW programme through training and implementation through their trainers, monthly continuous education sessions (generally called monthly refresher trainings) at catchment facilities, and monitoring of intervention delivery by programme supervisors and district health officials. The regular refresher sessions were based on the LHW programme's standard protocols and well documented.

Second, we used a detailed, independent data-collection system. We gathered data for outcomes and exposures through a quarterly active surveillance system via independent teams of data collectors. As part of the quality-assurance process, around 5% of the households were revisited by independent monitors within 3 days of the surveillance visits to corroborate findings. Trained programme managers also monitored LHW performance and activities with standard checklists and generated monthly summaries. Despite a few transfers of LHWs and some choosing not to participate, randomisation through the reporting facilities ensured no contamination between intervention and control clusters, although some sharing of messages between families and residents was inevitable.

Our trial also had several limitations in view of its scale and the fact that implementation was largely dependent upon LHW functionality and availability. There was a substantial loss of working days and suspension of routine and project-specific mother and child health-care intervention activities as a result of LHWs' deployment in periodic mass polio immunisation campaigns. LHWs were frequently tasked with additional duties for immunisation activities such as measles campaigns and child health days. During the massive seasonal floods of 2010 and 2011,^36^ LHWs were deployed twice for several months to provide flood relief activities in the district. We estimate that almost 30% of LHW time was spent on such activities during the trial. We do not regard this as improper implementation; rather, we think it reflects the reality of busy public sector programmes and employees, who have to multitask. Attendance of childbirths by LHWs alongside traditional birth attendants, although considered feasible in the initial assessment and actively promoted, was a particular challenge. LHWs attended only a small proportion of home births. Stated barriers to attendance included the need to travel alone, restricted mobility at night, that these activities were additional to their routine activities, and the lack of remuneration by families (unlike traditional birth attendants, who get compensated directly by families).

Information obtained by data collectors on household practices was based on maternal recall and not validated through direct observations, and the possibility of some respondent bias and over-reporting of recommended health-care activities cannot be ruled out. Owing to low maternal literacy and social taboos, a large proportion of mothers did not remember or report the exact date of their last menstrual period, and the possibility of differential misclassification of miscarriages and stillbirths cannot be excluded either.

Our effectiveness trial included fewer newborn infants in the early neonatal period than did other efficacy trials^21^, ^22^, ^29^, ^34^ from the region, but was nonetheless associated with a significant reduction in neonatal mortality. The trial also showed that, notwithstanding several limitations, some LHWs could establish rapports with and work alongside local traditional birth attendants, including attending some births. The reduction in neonatal deaths was associated with an increase in antenatal care visits and the use of clean delivery kits at birth. However, the proportions of births attended by LHWs (14%) and potentially asphyxiated newborns resuscitated by LHWs during those visits (4%) were very small. The same was true for early postnatal home visits by LHWs (25%) and provision of amoxicillin (16%) to overtly sick neonates in the intervention clusters. Although the rates were substantially higher in intervention than in control clusters, overall intervention coverage for these morbidities by LHWs in the intervention clusters remained very low and no differences in cause-specific mortality as assessed by verbal autopsies were identified, although the study was not powered to assess such effects. We therefore cannot ascribe improvements in neonatal outcomes to individual intervention components related to home-based treatment. The choice of oral amoxicillin for treatment of suspected pneumonia based on clinical features including fast breathing was consonant with WHO guidance at the time,^25^, ^26^ although this recommendation for early neonatal pneumonia or infection has been challenged,^37^ and criteria for presumed serious bacterial infections in neonates have become more holistic.^38^

A significant difference from previous findings in Sindh^15^ was the lack of effect on facility births and stillbirths. Although we trained public sector staff across all facilities in the district at the beginning of the trial, notable differences from Hala were identified in terms of levels of staff motivation, quality of care, and the availability of adequate around-the-clock services in public sector facilities and the district headquarter hospital. This finding is important in view of the general benefits of referral and care seeking noted in community-based interventions for maternal and newborn care.^20^ Previous studies have shown that care for preterm and low-birthweight infants and newborn resuscitation can be delivered in home settings by motivated and well-trained community health workers,^39^ but outside of skin-to-skin care,^29^ few have shown effective management of preterm infants in domiciliary settings.

We firmly believe that if packages of community-based maternal and newborn care are delivered through community health workers, appropriate measures to strengthen health systems, transport systems, and quality of care therein are also needed. In addition to community mobilisation and support, urgent attention is needed for the provision of adequate basic and emergency newborn care facilities in health facilities, accompanied by strengthening of quality care for preterm infants and infants with presumed serious bacterial infections.^40^, ^41^ Although the importance of community-based maternal and newborn care is well recognised, the limitations of this delivery platform in reducing neonatal mortality is also well appreciated. Growing evidence supports the provision of high-quality basic and emergency maternal and newborn care in referral facilities. Pakistan and other south Asian countries should prioritise both community-based strategies for promotive and preventive care and high-quality facility-based care as key measures to achieve universal health care and the Sustainable Development Goal 3 targets for maternal and newborn health.^8^
