# Supplementary material for: Effect of provision of home-based curative health services by public sector health-care providers on neonatal survival: a community-based cluster-randomised trial in rural Pakistan
Source: Lancet Glob Health. 2017 Jul 14;5(8):e796–806. doi: 10.1016/S2214-109X(17)30248-6 (PMC5762815; doi:10.1016/S2214-109X(17)30248-6)
Supplement: Supplementary appendix [file mmc1.pdf]

THE LANCET
Global Health

Supplementary appendix

This appendix formed part of the original submission and has been peer reviewed. We post it as supplied by the authors.

Supplement to: Soofi S, Cousens S, Turab A, et al. Effect of provision of home-based curative health services by public sector health-care providers on neonatal survival: a community-based cluster-randomised trial in rural Pakistan. *Lancet Glob Health* 2017; **5:** e796–806.

Web Appendix

Web figures (page 2-5)

Web Tables (page 6-12)

Web Figure 1

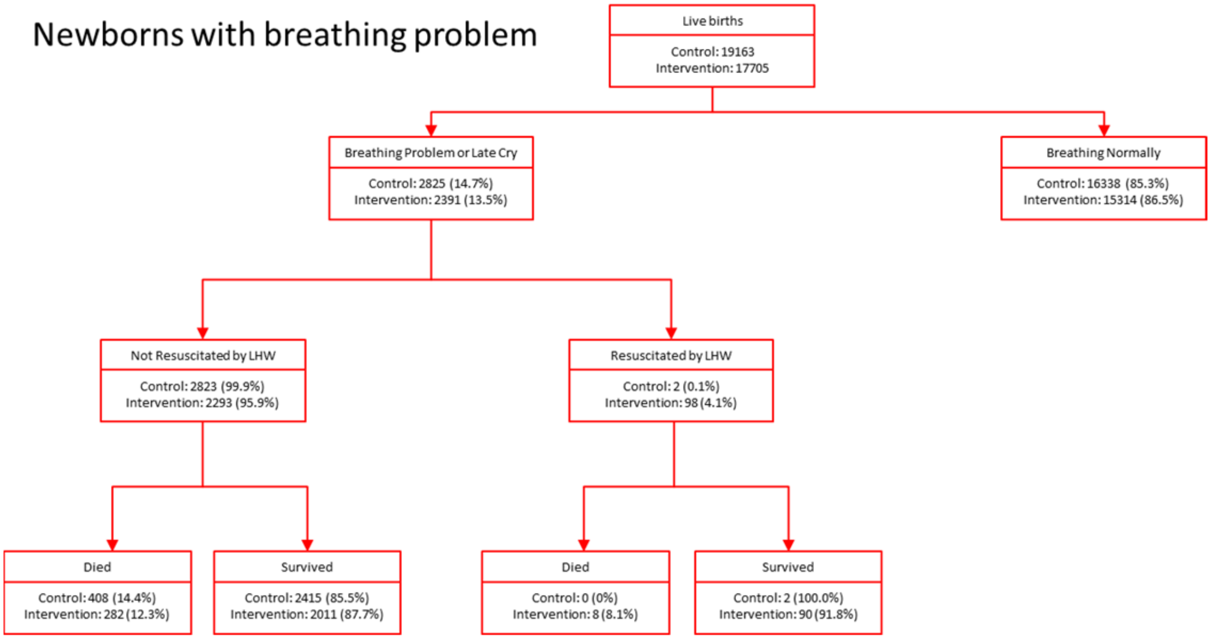
Newborns with breathing problem
Live births
Control: 19163
Intervention: 17705
Breathing Problem or Late Cry
Control: 2825 (14.7%)
Intervention: 2391 (13.5%)
Breathing Normally
Control: 16338 (85.3%)
Intervention: 15314 (86.5%)
Not Resuscitated by LHW
Control: 2823 (99.9%)
Intervention: 2293 (95.9%)
Resuscitated by LHW
Control: 2 (0.1%)
Intervention: 98 (4.1%)
Died
Control: 408 (14.4%)
Intervention: 282 (12.3%)
Survived
Control: 2415 (85.5%)
Intervention: 2011 (87.7%)
Died
Control: 0 (0%)
Intervention: 8 (8.1%)
Survived
Control: 2 (100.0%)
Intervention: 90 (91.8%)

Web Figure 2

Neonates with possible infection

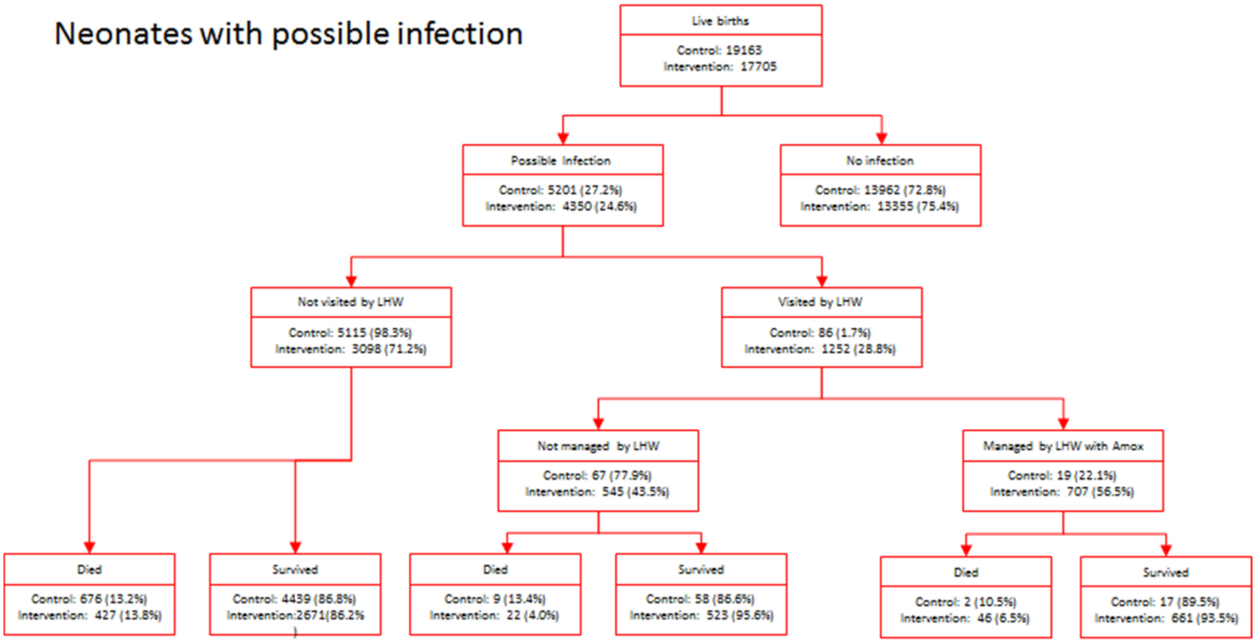

Web Figure 3

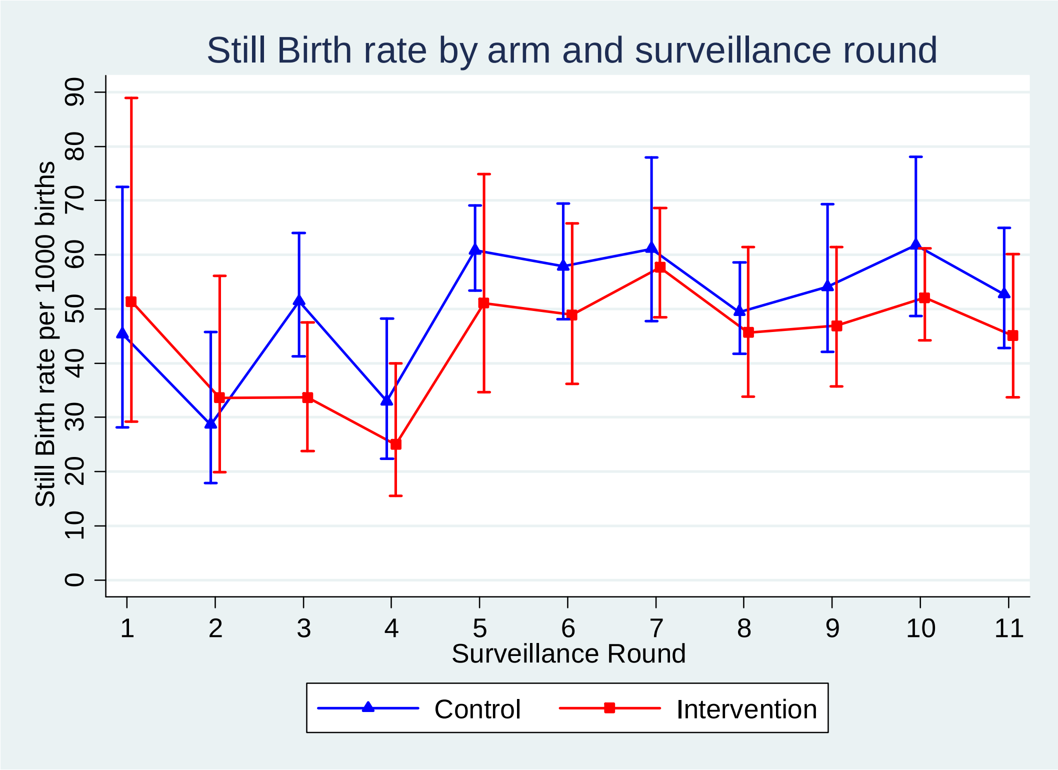
Still Birth rate by arm and surveillance round
Still Birth rate per 1000 births
0
10
20
30
40
50
60
70
80
90
1
2
3
4
5
6
7
8
9
10
11
Surveillance Round
Control
Intervention

Web Figure 4

Neonatal Mortality Rate by arm and surveillance round

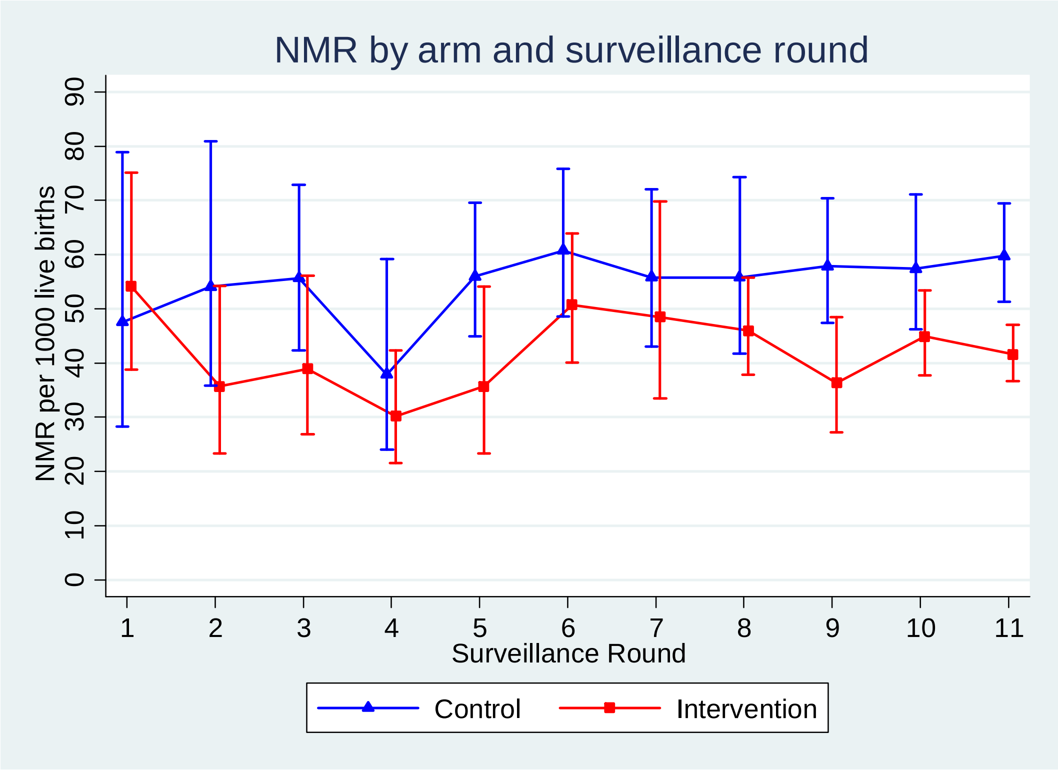

Web Table 1

Causes of Still births at Baseline

**Cause of Still Birth**	**Control (n=318)**		**Intervention (n=289)**	
	**n**	**Cause Specific NMR (95% ci)**	**n**	**Cause Specific NMR (95% ci)**
Congenital malformations	12	1.4 (0.6-2.2)	13	1.6 (0.7-2.5)
Pregnancy-induced hypertension	98	11.6 (9.3-13.9)	89	10.0 (8.7-13.3)
Antepartum haemorrhage	25	29.5 (18.0-41.1)	28	34.6 (21.8-47.4)
Maternal infections that can affect the foetus	21	2.5 (1.4-3.5)	17	2.1 (11.0-40.0)
Obstructed labour & Other obstetric complications	36	42.5 (28.7-56.4)	40	49.4 (34.1-64.7)
Other Causes	21	2.5 (1.4-3.5)	20	2.5 (1.4-3.5)
Cause not possible to determine from verbal autopsy	105		82	

Web Table 2
Cause Specific Neonatal Mortality at Baseline

Cause of Death	Control (n=329)		Intervention (n=306)	
	n	Cause Specific NMR (95% ci)	n	Cause Specific NMR (95% ci)
Preterm birth complications	63	7.8 (5.8-9.7))	61	7.9 (5.9-9.8)
Intrapartum complications (Birth asphyxia)	139	17.2 (14.3-20.0)	133	17.1 (14.2-20.0)
Neonatal Infections	103	12.7 (10.3-15.2)	95	12.3 (9.7-14.7)
Neonatal pneumonia	12	--	10	--
Neonatal sepsis/Meningitis	80	--	75	--
Neonatal tetanus	2	--	3	--
Neonatal diarrhoea	2 (0.6)	--	4 (1.3)	--
Congenital malformations	9	1.1 (0.3-1.8)	7	0.9 (0.2-1.5)
Accidents/Injuries	2	--	1	--
Other specific perinatal causes	7	--	3	--
Cause not possible to determine from VA	13	--	9	--

Webappendix Table 3

Cluster level summary analysis for birth outcomes

**Measure**	**GEE with round (as currently reported)**			**GEE without round**			**Cluster-level summary analysis (weighted by cluster size)**		
	**RR**	**95% c.i.**	**P-value**	**RR**	**95% c.i.**	**P-value**	**RR**	**95% c.i.**	**P-value**
Early fetal death	0.97	0.88, 1.08	P=0.60	0.97	0.87, 1.08	P=0.58	0.98	0.88, 1.10	P=0.80
Stillbirth	0.89	0.76, 1.04	P=0.13	0.89	0.76, 1.04	P=0.14	0.83	0.71, 0.98	P=0.04
Early neonatal mortality	0.79	0.67, 0.93	P=0.006	0.79	0.69, 0.93	P=0.005	0.77	0.63, 0.94	P=0.02
Late neonatal mortality	0.72	0.62, 0.85	P=0.0001	0.73	0.62, 0.86	P=0.0001	Number of late deaths very small in many clusters with several clusters having 0 deaths at either baseline or endline, preventing taking logs		
Neonatal mortality	0.80	0.68, 0.93	P=0.005	0.80	0.68, 0.94	P=0.005	0.77	0.64, 0.93	P=0.01
Perinatal mortality	0.86	0.75, 0.98	P=0.03	0.85	0.74, 0.98	P=0.03	0.80	0.69, 0.93	P=0.009

Web Table 4
Cause Specific Neonatal Mortality during Surveillance Rounds

Cause of Death	Control (n=934)		Intervention (n=653)		Cause Specific Mortality risk Ratio* (95% CI)	P values
	n	Cause Specific NMR† (95% ci)	n	Cause Specific NMR† (95% ci)		
Preterm birth complications	180	9.4 (7.4-11.4)	133	7.5 (6.3-8.7)	0.82 (0.67-1.01)	0.066
Perinatal asphyxia	394	20.6 (17.1-24.0)	257	14.5 (9.6-19.5)	0.81 (0.62-1.11)	0.10
Neonatal Infections	286	14.9 (12.2-17.6)	214	12.1 (9.0-15.1)	0.79 (0.60-1.05)	0.11
Neonatal pneumonia	*37*	--	*34*	--	--	
Neonatal sepsis/Meningitis	*248*	--	176	--	--	
Neonatal tetanus	*1*	--	*2*	--	--	
Neonatal diarrhea	*0*	--	*2*	--	--	
Congenital malformations**	23	1.2 (0.7-1.7)	17	1.0 (0.4-1.5)	0.81 (0.43-1.55)	0.53
Other specific perinatal causes**	17	0.9 (0.4-1.4)	12	0.7 (0.4-1.0)	0.88 (0.49-1.60)	0.69
Cause not possible to determine from VA	34	--	20	--	--	

† Cause Specific deaths per 1000 live births

* Estimated using generalised estimating equations, controlling for baseline mortality, randomisation stratum and surveillance round
** Estimated using generalised estimating equations, controlling for baseline mortality and randomisation stratum

Web Table 5
Causes of Still Births identified during Trial from Surveillance

Cause of Still Birth	Control (n=925)		Intervention (n=710)		Cause Specific Mortality risk Ratio* (95% CI)	P values
	n	Cause Specific NMR† (95% ci)	n	Cause Specific NMR† (95% ci)		
Congenital malformations**	30	1.5 (1.0-2.0)	24	1.3 (0.8-1.8)	0.93 (0.61-1.42)	0.75
Pregnancy-induced hypertension	277	13.7 (11.4-16.0)	221	11.9 (8.8-15.0)	0.87 (0.68-1.12)	0.28
Antepartum haemorrhage**	66	3.3 (2.5-4.0)	65	3.5 (2.8-4.2)	1.19 (0.99-1.44)	0.06
Maternal infections that can affect the foetus	74	3.6 (2.3-5.0)	44	2.4 (1.8-2.9)	0.69 (0.47-1.01)	0.06
Obstructed labour & Other obstetric complications	156	7.7 (5.5-9.9)	95	5.1 (3.7-6.5)	0.65 (0.49-0.86)	0.003
Other Causes**	34	1.7 (1.2-2.1)	25	1.3 (0.5-2.1)	0.78 (0.46-1.37)	0.40
Cause not possible to determine from verbal autopsy	288		236			

† Cause Specific deaths per 1000 births

* Estimated using generalised estimating equations, controlling for baseline mortality, randomisation stratum and surveillance round
** Estimated using generalised estimating equations, controlling for baseline mortality and randomisation stratum

Web Table 6

Post intervention Birthing practices and Neonatal Morbidity in the intervention clusters (with the additional analysis restricted to functional LHWs and their catchment households only)
(Excluding uncovered areas of Intervention ARM)

	Control clusters		Intervention clusters		OR (95% c.i); p-value
	n/N	%	n/N	%	
Home Births	5265/11262	46.7%	4847 /10248	47.3%	1.03 (0.82,1.30); P=0.78
Facility births	5997/11262	53.2%	5401/10248	52.7%	0.97 (0.77,1.21); P=0.78
Skilled birth attendant	6240/11262	55.4%	5512/10248	53.8%	0.92 (0.74, 1.16); P=0.51
Presence of LHW at deliveries conducted by TBAs	55/5022	1.1%	1020/4736	21.5%	25.20 (15.48, 41.01); P<0.0001
Instrumental deliveries	1879/11262	16.7%	1730/10248	16.9%	1.01 (0.82, 1.23); P=0.95
Use of CDK at home births	957/4492	21.3%	2983/4385	68.0%	9.97 (4.70, 21.15);P<0.0001
LHW postnatal visits	268/10496	2.5%	4431/9719	45.6%	33.57 (19.98, 56.42); P<0.0001
LHW early postnatal visits	185/10496	1.8%	3685/9719	37.9%	35.62 (21.81, 58.17); P<0.0001
Newborns with reported breathing problem or delayed cry at birth	1584/10801	14.7%	1342/9941	13.5%	0.90 (0.73, 1.12); P=0.35
Newborns with breathing problem resuscitated by LHW	1/1584	0.1%	83/1342	6.1%	110.06 (20.79, 582.73); P<0.0001
Low birth weight births	547/5774	9.5%	553/5992	9.2%	0.97 (0.77, 1.22); P=0.80
Neonates with reported illness	3747/10801	34.7%	3071/9941	30.9%	0.83 (0.63, 1.11); P=0.22
Care seeking for neonates with reported illness	3464/3747	92.5%	2844/3071	92.6%	1.06 (0.76, 1.47); P=0.72
Sick neonates visited by LHW	101/3747	2.7%	1269/3071	41.3%	25.12 (15.42, 40.93); P<0.0001
Neonates with possible infection	2928/10801	27.1%	2446/9941	24.6%	0.88 (0.67, 1.15); P=0.36
Neonates with possible infection seen by LHW	85/2928	2.9%	1011/2446	41.3%	23.46 (15.07, 36.52); P<0.0001
Neonates with possible infection managed by LHW using Amoxicillin	19/2928	0.6%	568/2446	23.2%	45.56 (22.31, 93.04): P<0.0001

LHW=Lady health worker

Web Table 7

Summary birth outcomes from the quarterly surveillance (rounds 1-11) (with the additional analysis restricted to functional LHWs and their catchment households only)
(Excluding uncovered areas of Intervention ARM)

	Control Clusters	Intervention clusters	Mortality risk Ratio* (95% CI)	p-value
Early Pregnancy Loss				
Number	746	642		
Rate per 1000 known pregnancies	62	59	0.96 (0.86, 1.07)	0.50
Still Births				
Number	608	427		
Rate per 1000 total births	53	41	0.79 (0.64, 0.97)	0.024
Early Neonatal mortality				
Number	481	329		
Rate per 1000 live births	44	33	0.80 (0.64, 1.00)	0.053
Late Neonatal mortality				
Number	103	58		
Rate per 1000 live births	10	6	0.57 (0.40, 0.81)	0.002
Neonatal mortality				
Number	584	387		
Rate per 1000 live births	54	39	0.78 (0.62, 0.99)	0.040
Perinatal mortality				
Number	1089	756		
Rate per 1000 total births	95	73	0.81 (0.65, 0.99)	0.044

* Estimated using generalised estimating equations, controlling for baseline mortality, randomisation stratum and surveillance round.

Web Table 8

Post intervention Birthing practices and Neonatal Morbidity in the intervention clusters
(Restricted analysis Comparing Overall Intervention Clusters with just LHW Covered Intervention Clusters)

	Overall Intervention Clusters		LHW Covered Intervention Clusters		OR (95% c.i); p-value
	n/N	%	n/N	%	
Home Births	8627/18325	47.1%	4847/10248	47.3%	1.01 (0.95,1.08); P=0.71
Facility births	9698/18325	52.9%	5401/10248	52.7%	0.99 (0.93, 1.05); P=0.71
Skilled birth attendant	9900/18325	54.0%	5512/10248	53.8%	0.99 (0.93, 1.05); P=0.67
Presence of LHW at deliveries conducted by TBAs	1184/8425	14.1%	1020/4736	21.5%	1.66 (1.40, 1.96); P<0.0001
Instrumental deliveries	3008/18325	16.4%	1780/10248	16.9%	1.03 (0.92, 1.14); P=0.64
Use of CDK at home births	4236/7698	55.0%	2983/4385	68.0%	1.75 (1.50, 2.05);P<0.0001
LHW postnatal visits	5256/17288	30.4%	4431/9719	45.6%	1.95 (1.62, 2.33); P<0.0001
LHW early postnatal visits	4318/17288	25.0%	3685/9719	37.9%	1.86 (1.56, 2.21); P<0.0001
Newborns with reported breathing problem or delayed cry at birth	2391/17705	13.5%	1342/9941	13.5%	1.00 (0.96, 1.04); P=0.96
Newborns with breathing problem resuscitated by LHW	98/2391	4.1%	83/1342	6.2%	1.53 (1.21, 1.93); P<0.0001
Low birth weight births	933/10125	9.2%	553/5992	9.2%	1.00 (0.95 1.05); P=0.88
Neonates with reported illness	5439/17705	30.7%	3071/9941	30.9%	1.00 (0.95, 1.05); P=0.99
Care seeking for neonates with reported illness	5037/5439	92.6%	2844/3071	92.6%	1.01 (0.93, 1.09); P=0.88
Sick neonates visited by LHW	1566/5439	28.8%	1269/3071	41.3%	1.74 (1.47, 2.06); P<0.0001
Neonates with possible infection	4350/17705	24.6%	2446/9941	24.6%	1.00 (0.95, 1.04); P=0.84
Neonates with possible infection seen by LHW	1252/4350	28.8%	1011/2446	41.3%	1.74 (1.48, 2.04); P<0.0001
Neonates with possible infection managed by LHW using Amoxicillin	707/4350	16.6%	568/2446	23.2%	1.55 (1.34, 1.78): P<0.0001

Web Table 9

Summary birth outcomes from the quarterly surveillance (rounds 1-11)

(Restricted analysis Comparing Overall Intervention Clusters with just LHW Covered Intervention Clusters)

	Intervention clusters	LHW Covered Intervention clusters	Mortality risk Ratio* (95% CI)	p-value
Early Pregnancy Loss				
Number	1191	642		
Rate per 1000 known pregnancies	61	59	0.97 (0.91, 1.02)	0.25
Still Births				
Number	830	427		
Rate per 1000 total births	45	41	0.92 (0.85, 1.00)	0.05
Early Neonatal mortality				
Number	610	329		
Rate per 1000 live births	35	33	0.95 (0.85, 1.05)	0.33
Late Neonatal mortality				
Number	126	58		
Rate per 1000 live births	7	6	0.84 (0.64, 1.11)	0.22
Neonatal mortality				
Number	736	387		
Rate per 1000 live births	42	39	0.92 (0.82, 1.03)	0.13
Perinatal mortality				
Number	1440	756		
Rate per 1000 total births	78	73	0.92 (0.85, 1.00)	0.06

* Estimated using generalised estimating equations, controlling for baseline mortality, randomisation stratum and surveillance round.
